# The Impact of Hypoxia on Neutrophil Degranulation and Consequences for the Host

**DOI:** 10.3390/ijms21041183

**Published:** 2020-02-11

**Authors:** Katharine M. Lodge, Andrew S. Cowburn, Wei Li, Alison M. Condliffe

**Affiliations:** 1 Faculty of Medicine, National Heart and Lung Institute, Imperial College London, London SW3 6LY, UK; k.lodge@imperial.ac.uk (K.M.L.); a.cowburn@imperial.ac.uk (A.S.C.); 2 Department of Medicine, University of Cambridge, Cambridge CB2 0SP, UK; wl225@medschl.cam.ac.uk; 3 Department of Infection, Immunity and Cardiovascular Diseases, University of Sheffield, Sheffield S10 2RX, UK

**Keywords:** neutrophils, hypoxia, degranulation

## Abstract

Neutrophils are key effector cells of innate immunity, rapidly recruited to defend the host against invading pathogens. Neutrophils may kill pathogens intracellularly, following phagocytosis, or extracellularly, by degranulation and the release of neutrophil extracellular traps; all of these microbicidal strategies require the deployment of cytotoxic proteins and proteases, packaged during neutrophil development within cytoplasmic granules. Neutrophils operate in infected and inflamed tissues, which can be profoundly hypoxic. Neutrophilic infiltration of hypoxic tissues characterises a myriad of acute and chronic infectious and inflammatory diseases, and as well as potentially protecting the host from pathogens, neutrophil granule products have been implicated in causing collateral tissue damage in these scenarios. This review discusses the evidence for the enhanced secretion of destructive neutrophil granule contents observed in hypoxic environments and the potential mechanisms for this heightened granule exocytosis, highlighting implications for the host. Understanding the dichotomy of the beneficial and detrimental consequences of neutrophil degranulation in hypoxic environments is crucial to inform potential neutrophil-directed therapeutics in order to limit persistent, excessive, or inappropriate inflammation.

## 1. Introduction

The innate immune system, comprising physical barriers together with molecular and cellular components, provides constitutive or rapidly mobilised effectors to defend the body from invading pathogens. Once a micro-organism overcomes the initial anatomical barriers, for example, by gaining entry through a skin abrasion, the ensuing release of cytokines and chemicals from injured cells, in addition to pathogen-derived factors, rapidly recruits neutrophils from the circulation [1]. Neutrophils are equipped with a number of cytotoxic mechanisms designed to recognise and eliminate pathogens promptly, and hence play an early and vital role in host defence. A key component of this weaponry is the release of pre-formed membrane-bound granules, which are situated in the cytoplasm and packaged with multiple microbicidal proteins and proteases [2]. In order to affect pathogen killing, these granules can fuse with the pathogen-containing phagosome, releasing their toxic contents into the vacuole to destroy the ingested micro-organisms. Neutrophil granules can also fuse with the plasma membrane, secreting their arsenal of proteins extracellularly (degranulation); this may be in response to pathogens that cannot be internalised, or due to an overwhelming extracellular inflammatory milieu [3,4]. In addition, granule proteins can be released extracellularly in association with neutrophil extracellular traps (NETs) [5]. Although the primary function of granule exocytosis is host defence, the release of cytotoxic granules into the extracellular environment also has the potential to cause damage to nearby host tissue.

## 2. The Relevance of Hypoxia to Neutrophils

Under physiological conditions, circulating neutrophils encounter a wide range of oxygen tensions, from a pO_2_ of 13 kPa in the main arteries to 3 kPa in capillaries and venules [6]. Due to the limited oxygen diffusion capacity, normal tissue oxygenation can be considerably lower (physiological hypoxia), as demonstrated in striated muscle, colonic epithelium, and even the skin, despite the latter being exposed to ambient oxygen [7,8,9]. In certain situations, for example, impairment of pulmonary gas transfer due to lung diseases such as interstitial fibrosis or emphysema, systemic hypoxaemia further compromises oxygen delivery to tissues. Moreover, a significant amplification of this physiological tissue hypoxia often occurs in inflammation, infection, or ischaemia (pathological hypoxia) due to damaged vasculature, a high metabolic activity of pathogens and host cells, or a reduction in metabolic substrates, e.g., by compartmentalisation of infection [10]. Extremely low oxygen levels have been shown in numerous pathological situations, including pulmonary and skin infections, ulcers, inflammatory bowel disease, chronic bronchitis, and solid tumours [11,12,13,14,15,16]. Indeed, in a model of acute intestinal inflammation, neutrophils themselves markedly depleted local oxygen levels through nicotinamide adenine dinucleotide phosphate (NADPH) oxidase consumption of molecular oxygen [17]. Thus, neutrophils recruited in large numbers to these disease-associated tissues or trapped in areas of microcirculatory impairment are exposed to profound levels of hypoxia. As well as being a consequence of inflammation, hypoxia can drive inflammatory processes; increased vascular leak and endothelial damage, and impaired resolution of infection have been demonstrated in hypoxic mice [18,19,20] or following ischaemic injury (where the interrupted blood supply causes tissue hypoxia) in various organs [21], and increased circulating pro-inflammatory cytokines are detected in humans at high altitude, where hypoxia is secondary to reduced atmospheric oxygen levels [22].

## 3. Sensing of Hypoxia by Neutrophils

As frontline effectors of host defence, neutrophils need to function effectively in hypoxic environments; to enable this, they have the ability to sense surrounding oxygen tensions and respond by modulation of several key functions. One such adaptation is a heightened degranulation response under hypoxic conditions [23,24,25,26], which is the focus of this review. Neutrophils rely on oxygen sensing by prolyl and asparaginyl hydroxylase enzymes, which control the expression of the hypoxia-inducible factors (HIFs). HIFs belong to a group of basic helix-loop-helix-PER-ARNT-SIM (bHLH-PAS) proteins that respond to oxygen and other stresses. HIF functions as a heterodimeric transcription factor, consisting of a constitutively expressed beta unit HIF1β and an alpha subunit that is incredibly oxygen labile [27]. Currently, three alpha subunits have been identified (HIF-1α, HIF-2α, HIF-3α); the predominant subtype present in neutrophils is HIF-1α [28]. In an oxygen-rich environment “normoxia”, HIF is inactivated by post-transcriptional hydroxylation of conserved proline amino acid residues within the α subunit. This prolyl hydroxylation, mediated by prolyl-hydroxylases (PHDs), generates a high-affinity binding site for the von Hippel Lindau protein (pVHL), leading to polyubiquitination and degradation of HIF by the proteasome. There is a second tier of HIF regulation through the action of asparagine hydroxylase, known as the factor inhibiting HIF (FIH), which inactivates HIF transcription by preventing binding of the transcription co-factor p300. PHDs and FIH display an absolute requirement for dioxygen, Fe(II), and 2-oxoglutarate. Under hypoxia, the lack of dioxygen reduces hydroxylase activity, allowing stabilisation of HIF-α, which then translocates to the nucleus and binds HIFβ. The HIF heterodimer binds hypoxia response elements (HREs), thereby regulating the neutrophil’s response to local oxygen tensions by promoting the transcription of multiple hypoxia-responsive genes [29]. It has also been shown that pro-inflammatory agents, including TNF-α and several species of bacteria, can lead to the aberrant stability of HIF, even in normoxia (pseudo-hypoxia) [26,30,31]. Hence, neutrophils may be subject to a range of regulatory inputs able to modulate HIF activity at sites of hypoxic inflammation. As HIF-driven hypoxia-adaptation processes depend on the transcription of effectors, HIF-independent pathways may provide neutrophils with more rapid adaptability. For example, hypoxia has been shown to reduce neutrophil production of reactive oxygen species (ROS) due to the lack of available molecular oxygen [32], which may be relevant to the degranulation response as the accumulation of ROS can inhibit degranulation signalling mediators [33]. It is possible that hypoxia (de)stabilises proteins involved in signalling pathways independent of protein synthesis, e.g., by altering phosphorylation. In other cell lines, hypoxia regulated the phosphorylation status of the translational control protein mTOR (mammalian target of rapamycin) and its effectors [34], and AKT [35]. Hypoxia may also exert HIF-independent effects on the actin cytoskeleton, and hence granule trafficking. For example, in a breast cancer cell line, hypoxia rapidly induced recruitment of actin-bundling proteins to the plasma membrane, which mediated enhanced endocytic uptake in a manner independent of HIF transcription [36].

## 4. Neutrophil Granule Formation and Composition

Neutrophils contain a number of granule populations which are characterised by their different protein cargoes. The prevailing hypothesis for the formation of granule subtypes is that of “targeting by timing”, as proposed by Borregaard et al., whereby concomitantly formed proteins are directed simultaneously into granules [37]. Hence, different subtypes of granule are formed as protein expression changes during neutrophil maturation. Traditionally, certain archetypal proteins have been used to distinguish granule subsets, with cell fractionation and proteomic techniques revealing relatively distinct granule protein composition [38,39]. The first granules to be formed are the azurophil (primary) granules, identified by the presence of myeloperoxidase (MPO), followed sequentially by the formation of lactoferrin-rich specific (secondary) granules and then gelatinase (tertiary) granules, which contain abundant matrix metalloproteinase 9 (MMP-9). Specific and gelatinase granules also contain the membrane subunits of NADPH oxidase (p22*^phox^* and gp91*^phox^*), which are delivered to the NADPH oxidase complex to enable the production of ROS. More recently, ficolin-1-rich granules have been described, formed at a similar time to gelatinase granules [39]. Each granule subtype contains many additional peptides, proteins, and proteases to supplement the canonical proteins that identify them. During the final stage of granulopoeisis, secretory vesicles are formed via an endocytic route, incorporating plasma proteins and cell surface receptors, such as the bacterial formylated peptide (fMLP) receptor [40]. Granule classification is functionally important as there is a hierarchical release dependent on the potency of the neutrophil activation stimulus. However, in reality, granules represent a spectrum, and some proteins are present in more than one granule subtype.

## 5. Mechanisms of Neutrophil Degranulation

### 5.1. Receptors and Intracellular Signalling Pathways

In order to prevent inappropriate triggering within the circulation, neutrophils undergo a two-step activation process. An initial priming agent generates a pre-activated “primed” phenotype, which results in swift initiation and potent augmentation of cytotoxic responses if the neutrophil subsequently encounters an activating stimulus or environment, such as a bacterial focus of infection [41]. Hence, neutrophil granule exocytosis instigated by an activating stimulus, e.g., fMLP, is markedly enhanced by prior interaction with a priming agonist, e.g., granulocyte-macrophage colony-stimulating factor (GM-CSF), tumour necrosis factor α (TNF-α) or platelet-activating factor (PAF). Neutrophils express a wide variety of cell surface receptors: G protein-coupled receptors (GPCRs) [42], integrins [43], and Fc receptors [44] allow the neutrophil to interact with a wide range of stimuli to generate a degranulation response, although the signalling pathways are complex and have not been fully delineated. Receptor ligation initiates intracellular signalling cascades; the precise signalling pattern likely depends on the range, concentration, and context of external stimuli, and downstream signalling events may occur in parallel. However, these pathways converge on common degranulation effector mechanisms, principally cytoskeletal rearrangement, and granule/vesicle trafficking and membrane fusion.

GPCRs, which interact with agonists such as the chemokines, PAF, and fMLP, are heterotrimeric proteins that dissociate into Gα and Gβγ subunits upon receptor ligation; the majority of neutrophil signal transduction is thought to occur through the Gβγ fragment. Gβγ activates two parallel pathways: activation of phospholipase C (PLC) generates inositol-trisphosphate (IP_3_) from phosphatidylinositol-(4,5)-bisphosphate (PIP_2_), resulting in the release of calcium from intracellular stores, whereas activation of phosphoinositide 3-kinase γ (PI3Kγ) generates phosphatidylinositol-(3,4,5)-trisphosphate (PIP_3_) from PIP_2_, with subsequent signalling predominantly via the protein kinase AKT. Both the elevated calcium [45] and AKT activation [46] contribute to granule exocytosis, and pan-PI3K inhibition or genetic deletion of the AKT2 isoform have been shown to inhibit degranulation [46,47]. Integrins (adhesion receptors), Fc receptors (which recognise immunoglobulin-opsonised pathogens and immune complexes) and cytokine receptors (whose ligands include GM-CSF, TNF-α, and selected interleukins) signal through tyrosine kinases: phosphorylation signalling cascades promote cytoskeletal actin and microtubule polymerisation through localisation of scaffolding proteins and recruitment of further adaptor signalling proteins [48]. Similar to GPCR activation, ligation of these receptors induces a PLC-dependent rise in intracellular calcium, culminating in granule exocytosis; neutrophils from mice lacking PLCγ2 failed to degranulate following β2 integrin or Fc receptor stimulation [49]. Although signalling molecules important for distinct degranulation pathways have been identified, such as proline rich kinase 2 (Pyk2), which is required for integrin-induced granule exocytosis but not that of soluble agonists [50], there is a significant degree of receptor cross-talk. For example, fMLP (which ligates GPCRs) can also initiate Src tyrosine kinase-dependent degranulation [51], and downstream activation of Rho and Rab GTPases (which mediate granule transport to the plasma membrane) can be initiated by the GPCR Gα subunit or Gβγ-PI3K signalling, or by the Fc receptor tyrosine kinase cascade [52].

### 5.2. Vesicle Trafficking and Fusion Machinery

Granule trafficking to the target phagosomal or plasma membrane is predominantly controlled by Rho and Rab GTPases, which facilitate granule migration by orchestrating cytoskeletal rearrangement. The Rho GTPase, Rac2, is particularly important in human neutrophils, mediating actin dynamics (and hence degranulation) through the formation of free actin filament ends, which regulates actin polymerisation and assembly [53]. Although functional characterisation of individual Rabs and their effector molecules is currently limited, one well-described example is granule-associated Rab27a, which mediates actin depolymerisation, thereby allowing local access to the vesicle docking zone [54]. Vesicle-membrane tethering, docking, and fusion are then regulated by Munc family proteins and soluble NSF-attachment protein receptors (SNAREs) (see [55] for a comprehensive review of vesicular trafficking machinery). Ultimately, translocation and exocytosis of granules require a rise in intracellular calcium, which occurs in a biphasic fashion, initially released from intracellular stores and then entering from the extracellular space as the intracellular stores become depleted [56]. Although the exact effectors of calcium-dependent degranulation are currently poorly characterised, examples of proteins which are influenced by calcium transients and involved in degranulation include annexins [57] and the vesicle docking facilitator, Munc13-4 [58]. In the absence of calcium, calmodulin inhibits granule fusion with the plasma membrane by binding SNAREs to sterically hinder fusion pore formation [59]. Calcium also plays a role in neutrophil polarisation via regulation of AKT, Src tyrosine kinases, and Rho GTPases [60], and this modulation of the cytoskeleton may contribute to the control of degranulation by calcium transients.

### 5.3. Regulation of Granule Exocytosis

Granule subtypes are not mobilised equally, as granule exocytosis is regulated by both the external stimulus and the secretory machinery. Sequential control of granule subtypes is regulated by the strength of the degranulation stimulus and is tightly linked to the order of granule development, as the last granules to be formed are the most easily mobilised. Sengeløv et al. showed an in vivo hierarchy of granule subtype mobilisation, with a complete mobilisation of secretory vesicles, and 38%, 22%, and 7% release of gelatinase, specific and azurophil granules, respectively, from neutrophils in skin blister exudate, dependent on the intracellular calcium concentration, which was preserved on further stimulation with fMLP [43]. Regulation of exocytosis in this manner is logical, as the hierarchy of release correlates with granule protein function. Initial mobilisation of secretory vesicles is able to supply the neutrophil surface with the receptors required for transmigration without liberating histotoxic proteins, which could harm the vascular endothelium. Subsequently, the secretion of gelatinase and specific granules allows the release of MMPs, such as gelatinase and collagenase, which can degrade the extracellular matrix (ECM), enabling neutrophil transit through host tissues towards sites of infection. Only once neutrophils have reached these inflammatory areas, where pathogen numbers and inflammatory stimuli are at the highest concentrations, is the more potent cargo of the azurophil granules unleashed as these granules require the strongest stimulus for degranulation [45]. The precise secretory machinery associated with a particular granule is also linked to its classification and function. Neutrophils display a non-redundant role for Rac2 in the secretion of azurophil but not specific, or gelatinase granules [61] but only a subset of azurophil granules have the required secretory machinery to be released extracellularly: Monfregola et al. demonstrated that Rab27a (which is located predominantly on gelatinase and specific granules, with lesser localisation to azurophil granules) was necessary for secretory azurophilic granules to fuse with the plasma membrane but was not required for their fusion with the phagosome [62]. Hence, even the most powerful degranulation stimulus liberates only a proportion of the cellular azurophil granules to the extracellular space. Further regulation of differential granule fusion and mobilisation is mediated by Src tyrosine kinases and granule associated-SNAREs, whereby association with certain family members dictates whether a granule is directed to the phagosome or plasma membrane [3,63]. Thus, a number of differential secretory control mechanisms result in the majority of azurophil granules being preferentially targeted to the phagosome rather than the surface membrane, unlike specific and gelatinase granules.

## 6. Effect of Hypoxia on Protein Secretion from Non-Neutrophils

Hypoxia has been shown to modulate protein secretion from several cell types, including immune cells and endothelial cells. Steiner et al. showed that rats exposed to systemic hypoxia experienced a rapid (within minutes) and sustained increase in mast cell degranulation in vivo [64], and the hypoxic culture of mast cells demonstrated a HIF-independent increase in release of pre-stored histamine assessed at 3 h [65]. Conversely, TNF-α secretion was decreased, and transcriptomic analysis revealed the downregulation of Sec24A, which is implicated in cytokine transport from the endoplasmic reticulum to the Golgi. These findings perhaps explain why TNF-α secretion is reduced in this context, but the mechanism of enhanced histamine release was not elucidated. Gulliksson et al. also showed a differential secretion profile for mast cells when cultured under hypoxia for 24 h [66]; here, tryptase release (indicating degranulation) was unaffected, IL-6 secretion was enhanced by hypoxia alone, and IL-8 secretion was decreased under hypoxia in response to selected stimuli [66], though again no mechanism was identified. 

In eosinophils, Porter et al. showed that hypoxia increased the release of IL-8 but attenuated that of eosinophil-derived neurotoxin, with no change in mRNA levels even at 24 h [67]. The phenomenon of “piecemeal degranulation” has been described for both mast cells and eosinophils, whereby release of selected proteins or cytokines occurs via vesicle or vesiculo-tubular sequential emptying of granules [68]; it is possible that hypoxia may be able to access or modify this system of protein secretion. Further investigation by Porter et al. revealed that hypoxia promoted focal actin polymerisation in eosinophils, with changes seen as early as 1 h. As localised actin polymerisation is essential for vesicle fusion, it is tempting to speculate that hypoxic modulation of this process could enhance degranulation.

Hypoxia appears to have a more consistent effect on mediator secretion from macrophages, with several studies showing an increased release of TNF-α, IL-6, IL-8, and monocyte chemoattractant protein 1 (MCP-1) [69,70,71,72]. An increase in IL-8 mRNA under hypoxia was seen as early as 30 min with a subsequent increase in protein secretion observed at 2 h, but pharmacological HIF-1α stabilisation did not recapitulate the IL-8 mRNA induction seen under true hypoxia, indicating a likely HIF-independent mechanism [70]. In this and one other study, hypoxia rapidly activated the transcription factor, activating protein-1 (AP-1), which regulates IL-8 production [70,71]. AP-1 activity is modulated by changes in the intracellular redox state, and a chemically induced reducing environment (mimicking hypoxia) was shown to increase AP-1 DNA-binding in tumour cells [73]. Hence, hypoxic activation of transcription may occur independently of HIF in macrophages, but increased cytokine secretion at early timepoints still appears reliant on induction of protein synthesis. Context-dependent differences in macrophage degranulation have been identified, where hypoxia increased MCP-1 secretion from cultured alveolar but not peritoneal macrophages; here, a transient increase in hydrogen peroxide production was observed prior to protein secretion, although a causal link was not proven [72]. Differences in cell metabolism have also been identified—anoxia increased L-arginine consumption and arginase activity in wound-derived but not peritoneal macrophages, although TNF-α and IL-6 release was increased from both cell types [69]. 

Hypoxia can also modulate the secretion of proteins from certain non-immune cells. Endothelial cells contain exocytosable Weibel–Palade bodies (WPBs), which are organelles containing proteins implicated in vascular homeostasis and inflammation, including the von Willebrand factor and the neutrophil adhesion molecule, P-selectin. WPB exocytosis pathways are not fully elucidated but appear similar to the translocation and docking mechanisms of neutrophil granules, as WPBs are associated with Rab proteins (including Rab27a) and interact with Munc and SNARE vesicle docking proteins [74]. Hypoxia could augment the release of WPBs from cultured endothelial cells at 4 h, which was maintained in the presence of protein translation inhibitors, indicating the release of pre-formed protein [75,76]. The enhanced release was dependent on a hypoxia-induced rise in intracellular calcium and the formation of multiple secretion pores in the plasma membrane [76]. Another potential mechanism of enhanced WPB release under hypoxia is triggering of exocytosis by increased vascular endothelial growth factor (VEGF), which is a HIF target gene, although the role of HIF in WPB release has not been directly assessed [77].

## 7. Effect of Hypoxia on Neutrophil Degranulation

### 7.1. Hypoxia Enhances Neutrophil Degranulation

**Figure 1:**
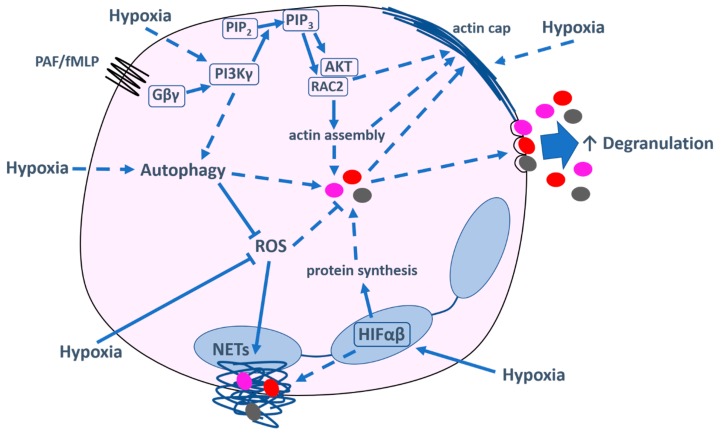
Mechanisms of hypoxia-enhanced neutrophil degranulation. Hypoxia increases neutrophil degranulation in a phosphoinositide 3-kinase γ (PI3Kγ)-dependent manner: activation of PI3Kγ by the G protein-coupled receptor βγ (G βγ) subunit induces the production of PIP_3_ by phosphorylation of PIP_2_. PIP_3_ signals via the AKT cascade and activates Rac2, which controls actin assembly. Hypoxia also induces the formation of focal “actin caps”. This rearrangement of the actin cytoskeleton likely facilitates granule translocation and fusion with the plasma membrane. Hypoxia and PI3K signalling increase autophagy, which enhances degranulation. Hypoxia and autophagy reduce reactive oxygen species (ROS) via lack of molecular oxygen and mitophagy, respectively; lack of ROS increases degranulation. Hypoxia stabilises hypoxia inducible factor (HIF)α, which dimerises with HIFβ in the nucleus. HIF heterodimer signalling increases the synthesis of granule proteins and may increase neutrophil extracellular trap (NET) production, which is predominantly ROS-dependent. Dashed lines indicate where pathways are uncertain or unknown.

Given the ability of hypoxia to alter protein secretion from various immune and non-immune cells, it seems relevant to assess the effect of hypoxia on neutrophils, which are highly secretory cells, frequently required to work in oxygen-deplete environments. We will focus on the effect of hypoxia on neutrophil degranulation (i.e., extracellular granule release) rather than granule fusion with the phagosome, as the latter is difficult to quantify and the effect(s) of hypoxia on this process are currently unknown. The overall consensus of the literature examining the effect of hypoxia on neutrophil degranulation is that hypoxia augments granule exocytosis, likely of all granule sub-types (see Figure 1 for summary diagram). Neutrophils isolated from healthy volunteers subjected to acute hypoxia showed increased neutrophil elastase (NE) release ex vivo [23], although these cells experienced ambient oxygen during isolation and on subsequent culture (“hypoxia-re-oxygenation”). Consistent with these results; however, isolated human neutrophils incubated under hypoxia released more NE, MPO, lactoferrin, MMP-8, and MMP-9 when compared with normoxia, representing increased secretion of azurophil, specific and gelatinase granules [24,25]. McGovern et al. and Hoenderdos et al. found that this hypoxia-augmented degranulation occurred rapidly (within 2 h), was not prevented by inhibition of protein translation, and could not be recapitulated by pharmacological HIF-1α stabilisation [24,32]. Together these data suggest that hypoxia drives an early increase in the release of pre-formed proteins and proteases in a manner that can be partially or totally HIF-independent, analogous to the increased release of histamine from mast cells [65] and WPBs from endothelial cells [75], but in contrast to IL-8 release from macrophages, which was HIF-independent but reliant on new protein synthesis [70]. Of interest, variable responses to different agonists have been seen in neutrophils, with both PAF and GM-CSF, but not TNF-α, able to promote hypoxic degranulation [24]. It is highly likely that hypoxic effects are environment and context-dependent, as observed for the differential secretion profile of alveolar versus peritoneal macrophages under hypoxia [72].

### 7.2. PI3K Signalling Is Involved in Neutrophil Degranulation under Hypoxia

Interrogation of degranulation signalling pathways has revealed an important role for PI3K signalling (Figure 1), as pre-treatment of neutrophils with either pan-PI3K or PI3Kγ (but not PI3Kδ) small molecule inhibitors prevented the hypoxic uplift of degranulation [24]. In contrast, restriction of calcium availability or inhibition of the PLC pathway reduced overall degranulation, but the hypoxic augmentation was maintained. PI3K signalling mediates a number of neutrophil functions, including chemotaxis [78] and survival [79], often with distinct roles for PI3Kγ and PI3Kδ [80]. Studies have shown inconsistent (increased, decreased, or unchanged) chemotactic responses of neutrophils to hypoxia [32,81,82], which are likely explained by variations in time, induction agent, and tissue site, and PI3K signalling has not been assessed in this context. Prolonged neutrophil survival under hypoxia is well established and has been shown, unlike degranulation, to be HIF- and NF-κB-dependent but PI3K-independent [83,84], albeit over a longer (20 h) timeframe. However, reflecting the results from degranulation studies, hypoxia-induced secretion of the survival factor, MIP-1β, was shown to be PI3K-dependent [83], although the role of HIF in this process was not assessed. How hypoxia might modulate PI3Kγ signalling in a HIF-independent fashion remains unclear. It is conceivable that hypoxic reorganisation of the actin cytoskeleton facilitates access of PI3Kγ to its phospholipid substrate; cytoskeletal disruption has been linked to enhanced neutrophil degranulation [85], and hypoxia has been shown to promote focal actin polymerisation in neutrophils, forming cap-like structures [24], similar to the actin reorganisation seen in hypoxic eosinophils [67]. Another possibility is that hypoxia is able to regulate PI3K expression. Studies in other cell types have demonstrated that hypoxia upregulates both PI3Kγ expression [86] and phosphorylation of its effector, AKT [87]. However, the fact that inhibition of protein translation in neutrophils did not prevent the early PI3Kγ-dependent hypoxic enhancement of degranulation argues against increased PI3Kγ expression mediating this effect.

### 7.3. Roles of Autophagy and Reactive Oxygen Species in Neutrophil Degranulation under Hypoxia

Further possible explanations for a HIF-independent hypoxic enhancement of degranulation involve autophagy and ROS production (Figure 1). Autophagy, the process by which damaged intracellular components are cleared by a homeostatic degradation pathway, promotes neutrophil degranulation—autophagy-deficient murine neutrophils exhibited reduced secretion of all three major granule subtypes (assessing MPO, lactoferrin, and MMP-9 release) [88]. Hypoxia has been shown to increase expression of LC3B-II, a protein indicative of autophagosome formation, in neutrophils, [89] and, hence, hypoxia-driven autophagy may contribute to increased degranulation. Furthermore, pan-PI3K inhibition reduced the formation of autophagosomes in hypoxic but not normoxic neutrophils, although this effect was only seen at 5 h (81). Conversely, a lack of ROS has been shown to enhance azurophil granule exocytosis in response to TNF-α and fMLP, as pharmacological inhibition of NADPH oxidase (which is responsible for ROS generation) enhanced NE release, an effect recapitulated using neutrophils from patients with chronic granulomatous disease (CGD) who have a defective NADPH oxidase complex [90]. Under hypoxia, ROS are reduced due to the lack of available molecular oxygen [32], and autophagy serves to prevent the accumulation of intracellular ROS by removing damaged mitochondria (mitophagy) and oxidised proteins [91]; these processes could work synergistically or independently to allow increased degranulation. However, as neutrophils rely predominantly on glycolytic metabolism, particularly in hypoxic situations [92], the contribution of mitophagy in this context is unclear. Complicating this picture further, autophagy defects in normoxic neutrophils led to decreased ROS production [88], and NADPH oxidase-generated ROS generation has been shown to be a key initiation signal for phagocytosis-dependent autophagy [93]. Both phagocytosis-dependent and -independent induction of autophagy have been shown, with uniform upregulation of LC3B and both processes reduced by PI3K inhibition; however, the reliance of phagocytosis-independent autophagy on ROS is less strict [94]. Of note, the aforementioned studies addressing neutrophil degranulation or hypoxia in association with autophagy demonstrated no differences in phagocytic capacity [88,89]. Overall, autophagy, which appears to increase degranulation, can be enhanced by both hypoxia and PI3K signalling, but the balance of interaction between autophagy and ROS in hypoxic situations, and the potential effect this has on degranulation, is complex and requires further clarification.

### 7.4. Role of HIF in Neutrophil Degranulation under Hypoxia

Examining a later degranulation response, Ong et al. demonstrated an increase in MMP-8 secretion from purified neutrophils after 24 h, both when cells were incubated under true hypoxia and when HIF-1α was stabilised pharmacologically [25], suggesting that this delayed response is HIF-dependent. It should be noted that the degree of neutrophil apoptosis under normoxia versus hypoxia begins to diverge after 5 h in culture, with significantly improved cell survival under hypoxia beyond 20 h, which may contribute to observed changes in protein secretion at later time points [95]. However, in this study, the authors demonstrated no significant difference in cell viability between normoxic versus hypoxic neutrophils exposed to media from tuberculosis (TB)-infected monocytes, as stimulated cells exhibited prolonged survival, which was not further augmented by hypoxia [25]. Consistent with this work, three further studies have suggested HIF-dependent changes in mRNA or protein content of selected neutrophil granule proteins (Figure 1). The same group showed an increase in MMP-8 gene expression under hypoxia after 24 h, although MMP-9 and NE gene expression were unchanged [25], despite putative HREs being identified in the promotor regions of both genes [24]. Peyssonnaux et al. showed an increase in murine neutrophil cathelicidin-related antimicrobial peptide (CRAMP, analogous to the human-specific granule protein cathelicidin) mRNA and protein content under hypoxia [26]. Although the length of hypoxic incubation in this study was unclear, both mRNA and protein levels of CRAMP were increased in vHL-deficient cells (where constitutive HIF degradation is reduced) but were absent in HIF-1α-deficient cells. Furthermore, activity of NE and cathepsin G was increased in vHL-null murine neutrophils, with the opposite true of HIF-1α-null cells. Similarly, in an in vitro *Pseudomonas aeruginosa* model, pharmacological HIF-1α inhibition for 18 h reduced the secretion of murine neutrophil beta-defensins and CRAMP [96]. This body of work suggests that longer-term exposure to hypoxia promotes a HIF-dependent increase secretion of selected proteins, which may be due to changes in granule protein content. However, it is important to recollect that the majority of granule biosynthesis occurs in the bone marrow, which is a profoundly hypoxic environment and, therefore, the impact of systemic hypoxia and HIF manipulation on this process must be interpreted in this context. Indeed Fouret et al. showed that NE, MPO, and lactoferrin mRNA transcripts were tightly controlled during granulopoiesis and that mature circulating neutrophils did not transcribe or translate NE mRNA [97]. Overall, it seems likely that HIF-dependent mechanisms are relevant to neutrophils encountering protracted hypoxia at inflammatory sites (where neutrophil survival is prolonged), and may modify granule protein synthesis in this setting as well as in the bone marrow. Moreover, neutrophils may encounter pro-inflammatory mediators capable of stabilising HIF-1α and, although it is uncertain whether such stimuli act synergistically with hypoxia, it is important to recognise that HIF signalling may be promoted in mildly hypoxic or normoxic environments. As well as being physiologically relevant, these observations have a bearing on the findings of experiments utilising HIF manipulation versus true hypoxia. Regardless of the stabilisation process, it must be remembered that HIF-driven hypoxia-adaptation processes depend on the transcription of effectors, and a HIF-independent control of granule exocytosis would allow quicker adaptation in the rapidly changing inflammatory environments encountered by neutrophil “responders”.

### 7.5. Role of Neutrophil Extracellular Traps in Releasing Granule Proteins

In addition to the process of degranulation, granule proteins can be unleashed by neutrophil extracellular traps, whereby expulsions of decondensed chromatin, studded with granule-derived antimicrobial proteins and proteases, are released into the extracellular space [5]. This process is thought to be a host defence mechanism, capable of trapping and killing micro-organisms. The classical molecular pathway initiating NET release relies on ROS generation by the NADPH oxidase complex, and pharmacological inhibition of NADPH oxidase abrogates NET generation; similarly, neutrophils from CGD patients display impaired release of NETs [98]. However, early (within 10 min of the stimulus), ROS-independent NETosis (the process of NET generation) has also been described after exposure of neutrophils to *Leishmania* promastigotes [99]. There has been fairly limited investigation into the impact of hypoxia on NET production with variable results reported, depending on whether assays were performed under true hypoxia or with HIF manipulation, again emphasising that both HIF-dependent and HIF-independent mechanisms may have context-dependent relevance. McInturff et al. demonstrated reduced NETosis in response to pharmacological and genetic HIF-1α knockdown [100]. Likewise, Zinkernagel et al. showed increased bacterial killing with pharmacological stabilisation of HIF-1α, which was maintained in the presence of a phagocytosis inhibitor but abrogated by the addition of DNAse (to dismantle NETs), although there was no directly observed effect on NET production [101]. In an in vivo murine study of ischaemic stroke, hypoxia increased circulating chromatin, which the authors speculate could be due to enhanced NETosis [102]. However, although Völlger et al. showed increased NETosis with pharmacological HIF-1α stabilisation, which was dependent on the presence of ROS [103], the same group showed either no difference (in response to *S. aureus*) or a decrease (in response to the non-physiological agonist phorbol myristate acetate) in NET production under true hypoxia [104]. Reduced NETosis was also seen in two other studies after hypoxic neutrophil culture, in response to bacteria or pro-inflammatory agonists [24,25]. As NETosis appears predominantly dependent on ROS generation, and hence reliant on oxygen availability, it is logical that NETosis under true hypoxia would be curtailed, although it appears that HIF-1α signalling also has a role to play. The complex in vivo interplay of these linked but opposing influences remains to be elucidated, and whether and to what degree NETosis contributes to granule protein release under hypoxia is currently uncertain.

## 8. Relevance of Hypoxia and Neutrophil Degranulation to the Disease States

### 8.1. The Role of Neutrophil Degranulation in Host Defence

Neutrophils are superbly equipped to carry out their role of host protection, with the ability to appropriately mobilise their antimicrobial granule contents being an important component of their defensive armamentarium. This is well illustrated by the increased susceptibility to infection of patients with neutrophil granule defects: patients with rare genetic mutations resulting in specific granule deficiency, or Chediak–Higashi syndrome (where neutrophils lack both cathepsin G and NE) suffer severe recurrent, predominantly bacterial, infections, despite preserved phagocytosis, and ROS production [105,106]. Neutrophils from patients with cystic fibrosis, who are vulnerable to repeated respiratory infections and bacterial colonisation, have decreased ability to kill bacteria, reduced Rab27a activity (which directs granules to the plasma membrane), and impaired degranulation of specific and gelatinase granules [107]. The impairment in Rab27a trafficking is thought to be due to defective ion transport by the cystic fibrosis transmembrane conductance regulator (CFTR). Treatment with the CFTR potentiator, ivacaftor, could restore Rab27a activity, increase degranulation and improve bacterial killing in vitro [107], and follow up of patients taking this treatment demonstrated reduced infection burden [108]. It is challenging to establish the extent to which extracellular granule release (rather than granule fusion with the phagosome) contributes to microbial killing *in vivo*, and studies of neutrophil supernatant-induced bacterial killing in vitro have shown variable results, dependent on the inciting stimulus and bacterial species [109,110]. However, in addition to their likely antimicrobial agenda, secretion of neutrophil granule products allows the communication with other immune cells—defensins and cathelicidin induced CD4+ and CD8+ T cell chemotaxis [111]; release of cathelicidin and azurocidin stimulated early recruitment and activation of inflammatory monocytes, resulting in increased bacterial clearance which was markedly impaired in neutropaenic mice [112]; and liberated serine proteases can modulate the activity of various cytokines [110]. These attributes confer a more complex and plastic role for neutrophils than previously recognised, and contribute to the crucial neutrophil function of the host defence.

### 8.2. Hypoxia-Driven Neutrophil Degranulation May Be Beneficial in Infection and Inflammation

**Figure 2:**
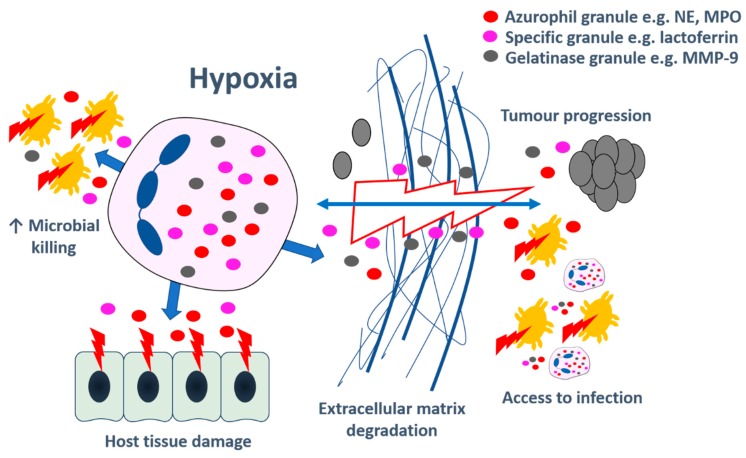
Consequences of hypoxia-enhanced neutrophil degranulation. Hypoxia increases the degranulation of azurophil, specific and gelatinase granules from neutrophils, which releases granule contents (e.g., neutrophil elastase (NE), myeloperoxidase (MPO), lactoferrin, and matrix metalloproteinase 9 (MMP-9)) into the extracellular space. Augmented release of these proteins and proteases may have beneficial effects: improved access to areas of infection through the extracellular matrix (double-headed arrow) and increased pathogen killing (damage indicated by lightning strike), or detrimental effects: host tissue damage, tumour growth and metastasis (double-headed arrow), and perpetuation of inflammation.

Neutrophils are required to combat pathogens in profoundly hypoxic areas of infection, inflammation, and microcirculatory impairment. The increased degranulation observed in hypoxic environments may contribute to the neutrophil’s success in clearing pathogens (Figure 2). For example, Pyk2 deficient mice (where neutrophil degranulation is impaired) were unable to effectively clear *Staphylococcus aureus* in a skin abscess model [50]; NE-deficient mice had substantially increased susceptibility to pneumonia [113] and gastrointestinal infection [114], and were shown to develop worse ventilator-induced lung injury [115], and mice lacking myeloid HIF-1α developed larger necrotic skin lesions with higher bacterial load following subcutaneous inoculation of group A *Streptococcus* [26]. Furthermore, increased secretion of certain neutrophil granule products may aid cross-talk with other immune cell types: cultured macrophages were able to ingest neutrophil granule proteins, which had a bacteriostatic effect on intracellular mycobacteria, suggesting that neutrophil granule exocytosis may lead to a co-operative defence strategy with other immune cells [116]. Another potential benefit of increased degranulation under hypoxia is improved access of neutrophils to sites of inflammation due to the release of more ECM-degrading granule products, e.g., MMPs. This enhanced degradation has also been shown to be instrumental in allowing revascularisation and implantation of transplanted tissue–hypoxia-induced VEGF-A signalling in a mouse model of avascular transplanted pancreatic islets recruited a subset of pro-angiogenic MMP-9^hi^ neutrophils, which enabled rapid revascularisation, with impaired angiogenesis seen in MMP-9-deficient mice [117].

### 8.3. Hypoxia-Enhanced Neutrophil Degranulation Can Damage Host Tissue in Acute Inflammation

Despite the potential benefits, increased degranulation under hypoxia is a double-edged sword as, in addition to enhanced antimicrobial and revascularisation potential, there is increased capacity for toxic granule products to cause extensive bystander tissue injury (Figure 2). Multiple infectious agents have been shown to induce hypoxia: Werth et al. demonstrated the upregulation of HIF-1α in infiltrating neutrophils in infected skin by several bacteria, viruses, and fungi in response to oxygen depletion [118]. Moreover, myeloid HIF-deficient mice had reduced leukocyte infiltration, MPO production, and oedema in a model of acute skin inflammation [119]. Hypoxia is a particular feature of abscesses, where replication of pathogens, massive infiltration of neutrophils, and the formation of a dense fibrin capsule to wall off the infection act in concert to deplete local oxygen levels [118]. In this profoundly hypoxic environment, both enhanced degranulation of destructive granule proteases, and release of toxic products due to bacteria-induced neutrophil lysis contribute to the host tissue breakdown and abscess formation (see Hajdamowicz et al. in this Special Issue for a comprehensive review [120]). Necrotic granulomatous regions in the lungs of TB-infected animals are severely hypoxic, demonstrated by hypoxia-probe staining and direct fibre optic tissue probe measurement of oxygen partial pressure [121]. Similarly, in humans, [18F]-misonidazole positron emission tomography (18F-MISO PET) revealed tissue hypoxia in areas of TB consolidation and cavitation [11]. Neutrophils are abundant in TB lesions [122], and hypoxia-enhanced release of neutrophil-derived MMPs resulted in substantially increased destruction of several ECM structural components in vitro [25]. In a zebrafish model, stabilisation of neutrophil HIF-1α led to a reduction in mycobacterial burden due to the enhanced formation of reactive nitrogen species [123], but these molecules are also able to cause local histotoxic effects at higher concentrations [124]. Together these results suggest that hypoxia may increase neutrophil capacity to drive tissue damage and cavitation in TB patients. 

Thus, what may initially commence as an entirely appropriate antimicrobial response can escalate into a highly damaging process, which may cause catastrophic injury to the host. This is exemplified by acute respiratory distress syndrome (ARDS), a heterogeneous lung injury with many precipitants, including bacterial pneumonia. Bilateral pulmonary infiltrates and hypoxia are defining criteria for the diagnosis of ARDS; increasing severity is indicated by worsening systemic hypoxia. Neutrophils sequestered in the lung rapidly accumulate in the alveolar space where gas exchange is substantially impaired due to oedema and alveolar collapse. Here, excessive neutrophil activation, including the degranulation of histotoxic proteins and pro-inflammatory cytokines, propagate diffuse host lung damage with high mortality [125]. In a murine model of hypoxia-induced acute lung injury, abrogation of hypoxic neutrophil survival by treatment with intra-tracheal IL-4 was able to promote resolution of inflammation and resulted in reduced bronchoalveolar lavage NE content, although the direct effect of IL-4 on degranulation was not assessed in this study [126]. Inhibition of glycolysis has also been shown to prevent neutrophil-driven tissue damage in murine acute lung injury [127]. Although neutrophils rely predominantly on glycolytic metabolism, even in the presence of oxygen, glycolytic flux can be further upregulated under hypoxia. Hypoxia-driven glycolysis provides neutrophils with a rapid energy supply, which enhances their motility, phagocytic capacity, and survival, and although the effect of these metabolism changes on degranulation has not been directly assessed, it seems highly likely that an increase in metabolic capacity would promote this process.

As well as having local tissue hypoxia, patients with ARDS are systemically hypoxic. Mice exposed to acute systemic hypoxia developed rapid onset morbidity and significantly increased mortality in response to staphylococcal skin infection and streptococcal pneumonia when compared with controls housed in normoxia [128]. This sickness behaviour was maintained in response to heat-killed bacteria or administration of LPS, implicating an exaggerated host reaction in perpetuating the systemic hypoxia-driven disease phenotype, which was found to be neutrophil-dependent. Intriguingly, hypoxic pre-conditioning protected the mice from the increase in morbidity and mortality observed with acute hypoxia, by suppression of HIF-driven neutrophil activation (although degranulation capacity was not assessed) and glucose utilisation. These key experiments highlight that hypoxic neutrophils can drive a highly detrimental systemic host response in addition to local tissue damage.

### 8.4. Detrimental Effects of Hypoxia-Enhanced Neutrophil Degranulation in Chronic Inflammatory Diseases and Cancer

As well as playing an important role in acute infection and inflammation, hypoxia is a significant factor in chronic inflammatory diseases. Direct measurement of synovial membrane oxygen tension in the inflamed joints of patients with rheumatoid arthritis (RA) during arthroscopy demonstrated extreme hypoxia [129]. Accumulation of neutrophils at the pannus-cartilage junction drives matrix degradation, and hence joint destruction, through the increased secretion of multiple proteases [130]. Notably, impaired degranulation from PLCγ2-deficient neutrophils afforded protection from the development of autoimmune arthritis in mice [49]. In the vasculature, neutrophils accumulate in atherosclerotic plaques, which are locally hypoxic, as shown by 18F-MISO PET and ex vivo immunohistochemistry. Enhanced release of toxic proteases from neutrophils has the potential to promote plaque progression and instability; for example, NE, detected in atherosclerotic lesions, was able to potentiate the processing and release of pro-inflammatory IL-1β from coronary endothelial cells [131] and promote endothelial apoptosis [132], and higher numbers of neutrophils were found in eroded or ruptured lesions [133]. In the lungs, HIF and pimonidazole staining indicate that bronchitic airways are severely hypoxic [134,135], which is relevant to several pulmonary diseases characterised by persistent neutrophilic inflammation, such as chronic obstructive pulmonary disease (COPD) and bronchiectasis. Damage to delicate lung tissue by excessive release of neutrophil granule contents in these pathologies has been well established: NE can cause lung epithelial damage and increase mucus secretion in vitro [24,136], induces the pathological features of COPD in animal models [137], and correlates with exacerbations and functional decline in bronchiectasis [138]. Of potential therapeutic interest, genetic depletion or pharmacological inhibition of PI3Kγ (implicated in the hypoxic enhancement of degranulation [24]), reduced lung tissue damage in a murine model of chronic bronchitis [139]. Local hypoxia is compounded by systemic hypoxaemia in severe lung disease; a common complication is the development of pulmonary hypertension (PH), where hypoxia and inflammation drive pulmonary vascular remodelling and intimal obstructive proliferation of distal pulmonary arteries [140]. Inhibition/depletion of the neutrophil degranulation products, NE or MPO, could prevent or reverse hypoxia-induced PH in rodent models [141,142]. 

Neutrophils have also been shown to contribute to cancer progression (Figure 2). They are abundant in tumours and can be either pro- or anti-tumorigenic [143]. Multiple tumour types have been shown to be markedly hypoxic [16], which may favour a pro-tumorigenic neutrophil phenotype—the increased release of neutrophil MMP-9 can promote cancer growth by enabling angiogenesis [144], whilst neutrophil proteases can facilitate tumour cell invasion by activating pro-MMP-2 [145]. Furthermore, the delivery of NE to adenocarcinoma cells in a murine lung cancer model induced tumour growth and increased mortality [146].

## 9. Conclusions

In summary, neutrophils play a vital role in protecting the host against invading pathogens, with degranulation of antimicrobial proteins and proteases being a key part of this defence. Areas of infection and inflammation are frequently severely hypoxic, and neutrophils operating in these environments are, therefore, subject to functional modulation by hypoxia. Hypoxia acts to increase neutrophil granule exocytosis, which may be an early HIF-independent process with activation of the PI3Kγ signalling pathway or a later response due to HIF-dependent gene transcription. Despite potential benefits of hypoxia-augmented degranulation, including improved neutrophil access to sites of infection and intensified pathogen clearance, there is substantially increased capacity for toxic granule products to cause both local tissue damage and systemic complications. By seeking a better understanding of the mechanisms by which hypoxia acts to control neutrophil degranulation responses, and the factors which determine a beneficial or detrimental host outcome, we will be better placed to identify new therapeutic strategies to combat neutrophil-driven morbidity in acute and chronic inflammatory disease.
